# Understanding HRH recruitment in post-conflict settings: an analysis of central-level policies and processes in Timor-Leste (1999–2018)

**DOI:** 10.1186/s12960-018-0325-5

**Published:** 2018-11-29

**Authors:** Maria Paola Bertone, Joao S. Martins, Sara M. Pereira, Tim Martineau, Alvaro Alonso-Garbayo

**Affiliations:** 1 ReBUILD Consortium & Institute for Global Health and Development, Queen Margaret University, Queen Margaret Drive, Edinburgh, United Kingdom; 2 ReBUILD Consortium & Faculdade de Medicina e Ciências da Saúde, Universidade Nacional Timor Lorosa’e, Dili, Timor-Leste; 3 ReBUILD Consortium & Liverpool School of Tropical Medicine, Liverpool, United Kingdom

**Keywords:** Human resources for health, Health workers, Recruitment, Deployment, Fragile and conflict-affected settings, Timor-Leste

## Abstract

**Background:**

Although human resources for health (HRH) represent a critical element for health systems, many countries still face acute HRH challenges. These challenges are compounded in conflict-affected settings where health needs are exacerbated and the health workforce is often decimated. A body of research has explored the issues of recruitment of health workers, but the literature is still scarce, in particular with reference to conflict-affected states. This study adds to that literature by exploring, from a central-level perspective, how the HRH recruitment policies changed in Timor-Leste (1999–2018), the drivers of change and their contribution to rebuilding an appropriate health workforce after conflict.

**Methods:**

This research adopts a retrospective, qualitative case study design based on 76 documents and 20 key informant interviews, covering a period of almost 20 years. Policy analysis, with elements of political economy analysis was conducted to explore the influence of actors and structural elements.

**Results:**

Our findings describe the main phases of HRH policy-making during the post-conflict period and explore how the main drivers of this trajectory shaped policy-making processes and outcomes. While initially the influence of international actors was prominent, the number and relevance of national actors, and resulting influence, later increased as aid dependency diminished. However, this created a fragmented institutional landscape with diverging agendas and lack of inter-sectoral coordination, to the detriment of the long-term strategic development of the health workforce and the health sector.

**Conclusions:**

The study provides critical insights to improve understanding of HRH policy development and effective practices in a post-conflict setting but also looking at the longer term evolution. An issue that emerges across the HRH policy-making phases is the difficulty of reconciling the technocratic with the social, cultural and political concerns. Additionally, while this study illuminates processes and dynamics at central level, further research is needed from the decentralised perspective on aspects, such as deployment, motivation and career paths, which are under-regulated at central level.

**Electronic supplementary material:**

The online version of this article (10.1186/s12960-018-0325-5) contains supplementary material, which is available to authorized users.

## Background

Human resources for health (HRH) represent a critical element for the functioning of health systems. Poor availability and management of human resources have been recognised as key health system barriers, and despite the efforts, many low-income countries continue to face acute HRH challenges [1, 2]. These challenges are compounded in conflict-affected settings where health needs are exacerbated as a result of the conflict and the health workforce is often decimated by either death or flight due to violence [3, 4]. Consequently, the challenge of attracting and recruiting the right persons into the health workforce, according to needs, becomes more acute and relevant [5–7]. Some studies in low and middle-income countries (LMICs) have focused on Human Resource Management (HRM) [8–14] and highlighted how recruitment affects retention in remote areas [15–19]. Research has also looked at the specific HRM challenges in fragile and conflict-affected countries [5–7, 20–27], with a focus on health worker retention and (financial and non-financial) incentives.

**Fig. 1:**
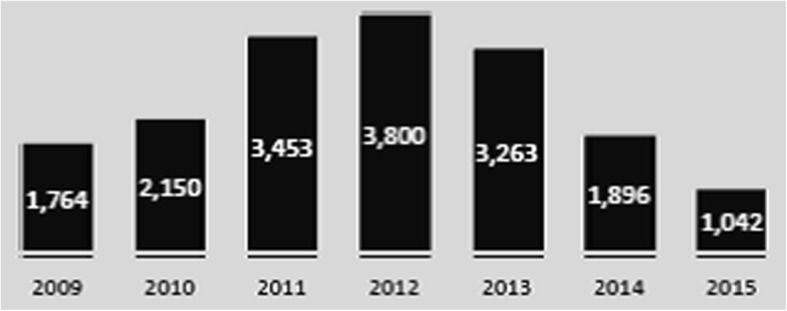
Oil and gas revenues in Timor-Leste (2009–2015) (US$ million). Source: [43]

This research adds to that literature by focusing in particular on the role that recruitment policies and practices play in rebuilding an appropriate health workforce after conflict and following the trajectory and patterns over the subsequent years. Our case study was conducted in Timor-Leste starting from the immediate post-conflict period up to 2018 (Table 1 and Fig. 1), which gives a retrospective view covering a period of almost 20 years. This paper focuses on policies and policy-making processes at central level related to health workforce recruitment and presents a political economy analysis about how and why both official and informal practices developed, as well as the drivers, challenges and blockages at different stages.

**Table 1:** History and context of Timor-Leste

The history of Timor-Leste has been one of struggle for self-determination. In 1975, after more than four centuries of colonial domination, Timor-Leste proclaimed its independence from Portugal. This lasted only 9 days before Indonesia illegally occupied the country. A repressive government ruled for 24 years (1975–1999) during which around 20% of the population died due to violence, starvation and disease [32]. The publication in September 1999 of the results of a UN-backed referendum to determine Timor’s status in favour of independence triggered a violent withdrawal of Indonesia which left around 1 400 people killed, more than 300 000 displaced and the governance structure and infrastructure including the health system virtually collapsed. In May 2002, the country finally restored its independence after a UN transitional government (United Nations Transitional Administration in East Timor—UNTAET) was put in place from 1999 to 2002 and facilitated the process of development of a new independent Timorese government [59, 60]. The UNTAET and World Bank-managed support to the transition was considered a successful development story [38]. Since independence, there have been four presidential elections (2002, 2007, 2012 and 2017) and a succession of eight Constitutional Governments, and the country ranked highest the Democracy Index of the Economist Intelligence Unit in South East Asia in 2016 [61]. However, an outbreak of violence in 2006–2007, triggered by disputes within the Armed Forces between the leadership and the ex-FALANTIL soldiers (armed group fighting in the independence struggle, which had been absorbed into the Army), caused the displacement of 150 000 people and led to the resignation of the Prime Minister. This wave of instability revealed the tensions between leaders, the frustration among the younger population due to high unemployment, and other state-threatening problems such as ethnic rivalry between the East and West of the country [62, 63]. The violent events impacted the health system, although most services remained available thanks to the Cuban Medical Brigade (CMB) which was perceived as a neutral health workforce and therefore not targeted [64]. However, the violence was reported to have had a negative impact on the perceived State legitimacy [65]. While no such level of violence has happened since, tensions still prevail and few people have perceived benefit from the positive development of macroeconomic indicators [66]. The country has actually greatly benefited from oil revenues since signing an agreement with Australia in March 2003 on the exploitation of the gas and oil fields in the Timor Sea [67]. However, the economic benefits of this have not reached all layers of society equitably, and in 2014, more than 41% of the population was still reported to live below the poverty line [68]. In addition, loans from World Bank, ADB and other bilateral and multilateral institutions appear to be largely invested in ‘megaprojects’ with limited impact on the overall economy. Cronyism and corruption are growing significantly [69] with limited local capacity and accountability being built over time in this regard, and as a result, the Corruption Perception Index increased from 28 in 2014 to 38 in 2017 [41]. Related to the violent events in 2006 and following a 14-fold increase in national budget from US$135M in 2006 to US$1 850M in 2013, mainly from oil revenues [70], the civil service grew by 75% from 20 000 to 35 000 staff [71]. This was reported to have mitigated the risk of further violence [71], but the increase in government’s capacity has not been commensurate with staffing growth; furthermore, the workforce expansion is unsustainable in light of the decline in oil revenues in more recent years (Fig. 1) [42, 43, 70]. Elections in July 2017 resulted in a minority government which lasted only a few months before the President dissolved the Parliament in January 2018 and called for early elections which were held in May 2018. Today, Timor-Leste has a population of 1 167 242 with 70% under the age of 35, an equal gender distribution and an estimated annual growth rate of 1.81% [72, 73].

## Methods

This study is part of a broader research covering HRH recruitment and deployment in Timor-Leste and exploring both the central and sub-national debates. In the present paper, we focus exclusively on the central-level perspective. The research adopts a retrospective, qualitative case study design [28], drawing from documentary review and key informant interviews. Policy analysis was conducted, with elements of political economy analysis which allowed the identification of how elements of the agency (actors, agendas, power relations) and structure (socio economic conditions, historical legacies, formal and informal institutions, cultural norms) contributed to drive the policy trajectory [29].

### Data collection

A document search was carried out to identify published literature and documents relevant to HRH recruitment policies and regulatory frameworks in Timor-Leste between 1999 and 2017. Internet searches were carried out using Google, PubMed, HRH Journal and Government websites (e.g. Official Gazette, Public Service Commission, UNTAET legislation archives). Additionally, key informants were asked to suggest relevant documentation, and the contextual knowledge of the team in country also allowed retrieving further documents. In total, 76 documents were reviewed (a summary is provided in Additional file 1).

Key informants were purposefully selected to include a wide range of actors involved in HRH policy-making over the study period. Twenty key informants were interviewed (Table 2) by AAG and JM between March and May 2018, either face-to-face or remotely, in Tetum, English, Spanish or Portuguese depending on the location and the language preference of the respondent. A standard topic guide was used flexibly to adapt to each respondent’s knowledge and elicit a retrospective enquiry about the main HRH challenges with a focus on recruitment, policy solutions to HRH issues, changes in policies and practices, drivers of change, actors and agendas (Additional file 2).

**Table 2:** Summary of key informants by type and gender

Type of KI	TOTAL	Gender	
		M	F
Ministry of Health (MoH)	8	8	0
National (governmental non-MoH)	5	5	0
International*	7	4	3
Total	20	17	3

*International actors include both those who were still in Timor-Leste at the time of the interviews as well as those who had been there some time during the study period but are now based elsewhere

### Data analysis

Information was extracted from the documents using an Excel-based template including an analytical thematic framework developed for this purpose (Additional file 3). Based on this, an initial draft of a ‘policy timeline’ was prepared which described in chronological order the key events and changes that had happened. The documentary analysis also helped to identify gaps in the information available, so that these could be addressed during the key informant interviews.

Key informant interviews (KII) were recorded, transcribed and translated into English (where needed). Verbatim transcripts were manually analysed, using a series of pre-defined descriptive themes (Additional file 3), but also focusing on emerging cross-cutting, analytical themes, more apt to capture the political economy issues and the dynamics which shaped the decision-making processes [30]. Data triangulation was carried out between different sources of data, comparing the narratives of different key informants, but also triangulating information from informants and documents. Based on the KIIs’ analysis, the policy timeline was revised, updated and enriched and key elements, themes and patterns were teased out and discussed among all authors before the final drafting of the article.

Ethical approval was obtained from the Liverpool School of Tropical Medicine and from Timor-Leste National Health Institute.

## Results

In this section, we present the main findings of our analysis following a chronological order to show how events unfolded overtime. We also highlight the key turning points and the main drivers and dynamics that shaped the policy-making processes.

### Immediate aftermath of Indonesia’s withdrawal and the transitional period (1999–2002)

In September 1999, with the violent withdrawal of Indonesia, the health system, like much of the rest of the social, economic and organisational structure and the infrastructure of Timor-Leste, had been destroyed [31]. In terms of HRH, the damages were profound. Most of the 135 doctors working in Timor-Leste under the Indonesian government were non-Timorese and left; only about 26 doctors remained [32]. Other critical staffing issues included the shortage of midwives, technicians and health managers; the rural-urban imbalances which were further exacerbated by the insecurity following the 1999 violence; and the excessive number of nurses inherited from the Indonesian system [33].

Faith-based and non-governmental organisations (NGOs) were the first to be able to provide basic health services [31, 34], and to do so, they brought in expatriate staff, but also recruited and paid local health personnel available in the districts [35]. Key informants reported that recruitment criteria were rather loose as it was not always possible to find formally qualified staff to fill the position and vacancy advertisement and recruitment were often based on word-of-mouth.

We did not do the recruitment per merit. We just indicated people. […] I called them to the health centre and proposed them to the expatriates of the NGOs. If they agree, we’d recruit them. (MoH KI)

While much of the technical and financial support for the health sector (including HRH) was provided by external partners [36], the Timorese health leaders of the nascent Ministry of Health (MoH) were keen to strengthen their role and increase the legitimacy of the state and the credibility of the emerging government through the so-called timorisation process [32, 33]. In line with this state-building aspiration, it was important for them to build a health workforce that would distance itself from the Indonesian one, considered inefficient. Key informants recalled:

The administrators took the line that it wasn’t just matter of putting people back into the positions they were in and paying them, but it was creating the whole civil service. […] They were very much trying to get a sense of unity [through the civil service]. (international KI)

This overhaul was to be achieved in three ways. Firstly, it was imperative to rationalise the number of health workers (nurses, in particular) employed in the public sector which was inflated for political and patronage reasons under the Indonesian occupation. Secondly, there was a desire to reduce or eliminate corruption, collusion and nepotism (often referred to using the Indonesian acronym, KKN) that was a key feature of the previous system and introduce a meritocratic approach. Finally, it was important to create a health workforce loyal to the new country, excluding those who had supported the pro-Indonesia militia during the independence struggle (KIIs). The aim was to ensure equitable access to quality service for the entire population of the newly formed state.

Our leaders wanted to build a public administration that was not a replica of the previous system. So, when we defined the regulations to form our public service, we did not look at the Indonesian model which was disproportionate. We started from zero. (national KI)

These objectives laid the foundation of the first civil service recruitment which started in mid-2001 (UNTAET Regulation 2000/03). The final estimate of the health staff needed was of 1 242 as agreed between UNTAET, MoH and the Civil Service and Public Employment Office (CISPE). Informants reported that this number, about 47% of the 2 632 in place during the occupation, was mainly dictated by the budget available, which was limited and dependant on international partners [37]. Fifty job descriptions were developed, vacancies opened, and recruitment of health staff began. In order to ensure staff retention in remote areas, vacancies were initially tied to specific locations rather than a central pool from which new recruits would subsequently be deployed (an approach which was later adopted). According to one key informant, in line with the aims of the recruitment, selection criteria included professional training, place of birth and perceived loyalty to the new state, against the Indonesian regime.

The first criteria was that the person had to be trained as a nurse or healthcare professional. The second, we had to look for people that were from that district to facilitate them going there […]. Then the third criteria that we considered was that it had to be people that in 1999 didn’t run away to Indonesia. These people are a priority. (MoH KI)

However, a number of issues hampered the recruitment process. The shortage of doctors remained a challenge and terms and conditions provided by NGOs were much more favourable so that many stayed in that sector. Additionally, strikes and protests broke out due to accusations of favouritism and mismanagement. One informant recalled:

The Bishop came down to calm the situation at the hospital and to listen to the demands of the demonstrators. They were saying that those who had fought for independence had not passed the recruitment. So, I went with [xxx] and we modified the recruitment. Those who had stayed would have work first, and those who went to Indonesia, they have to work last. (MoH KI)

In 2001, 724 workers were recruited (Table 3). The remaining vacancies were filled by international staff recruited by NGOs or by the MoH with Australian Government’s funds.

**Table 3:** Public workforce situation (October 2001)

	Establishment	Filled	Vacant	Vac. rate (%)
National level				
National DHS	49	22	27	55
NCHET	32	23	9	28
Central Lab.	22	15	7	32
National Hosp.	251	9	242	96
Districts				
Aileu	46	36	10	22
Ainaro	42	34	8	19
Baucau	152	135	17	11
Bobonaro	85	69	16	19
Covalima	58	46	12	21
Dili (District)	123	50	73	59
Ermera	54	38	16	30
Lautem	60	42	18	30
Liquica	39	38	1	3
Manatuto	48	40	8	17
Manufahi	57	44	13	23
Oecusse	57	29	28	49
Viqueque	67	54	13	19
TOTAL	1 242	724	518	42

Source: Civil Service and Public Employment Office (CISPE) 2001

*DHS* Division of Health Services, *NCHET* National Centre for Health Education and Training

Recruitment was reported to be slow and haphazard leading to poor morale and loss of trust in the administration by those involved in the process [32]. Their analysis lucidly highlights the trade-off between incompatible ‘technical’ and ‘political’ objectives (Table 4) [32]. However, despite the salience of the HRH challenges identified then, the immediate health sector reconstruction in Timor-Leste and the model of post-conflict health system rehabilitation has generally been judged a success [33, 38].

**Table 4:** Competing goals for the recruitment of civil servants for the health sector

Goal	Competing goal
• Produce measurable results quickly	• Achieve transition to full East Timorese management
• Disburse funds quickly	• Ensure national decision making and full ownership • Focus on building capacity • Ensure sustainability
• Ensure a coherent sector-wide approach	• Accommodate individual donor needs
• Provide services to all now	• Improve scope and quality of services
• Develop health policies soon, before it is ‘too late’	• Consult widely on all policy issues • Start flexible to avoid setting directions too early

Source: [32]

### A ‘game changer’: the involvement of the Cuban Medical Brigade (2003–2005)

The idea of requesting Cuban support to address staff shortages had been discussed since 2001 [39]. In February 2003, this option was adopted at top political level when an agreement was brokered between the then President Xanana Gusmao, José Ramos Horta (Minister of Foreign Affairs) and Fidel Castro for the provision of Cuban doctors as well as the training of Timorese students. The Cuban involvement was described by one key informant as ‘a game changer’ for the health sector. In December 2004, the engagement was further detailed as the training of 1 000 doctors (calculated as roughly 1 doctor per 1 000 population) at the Latin American Medical School (LAMS) in Cuba^1^ and the deployment of 300 Cuban doctors to provide medical care across the country as well as to supervise and further train the medical students and new doctors [39].

The use of subsidised training effectively shifted the focus from post-graduation recruitment procedures to pre-service education scholarship awards, as those trained in Cuba were expected to return to serve in Timor-Leste and were automatically absorbed in the public health sector.^2^ While in recent years, the absorption of medical graduates into public service is becoming problematic (see below), at the time, it was clear that scholarship assignment became a synonym of hiring.

Instead of recruiting, the MoH is appointing through training. (national KI)

The selection of students to access Cuban medical training was undertaken initially by the MoH, based on academic results in secondary school, a written exam, an interview and a physical examination (KIIs). Gender balance was also sought in the selection, and quotas were introduced based on the geographical origin of the candidates,^3^ with the intention that they would be deployed in their districts of origin ensuring an appropriate coverage across districts, increasing retention and health worker acceptability for local communities. Although the geographical criterion initially created difficulties due to the low skills of those coming from remote areas, these were overcome in subsequent years.

**Fig. 2:**
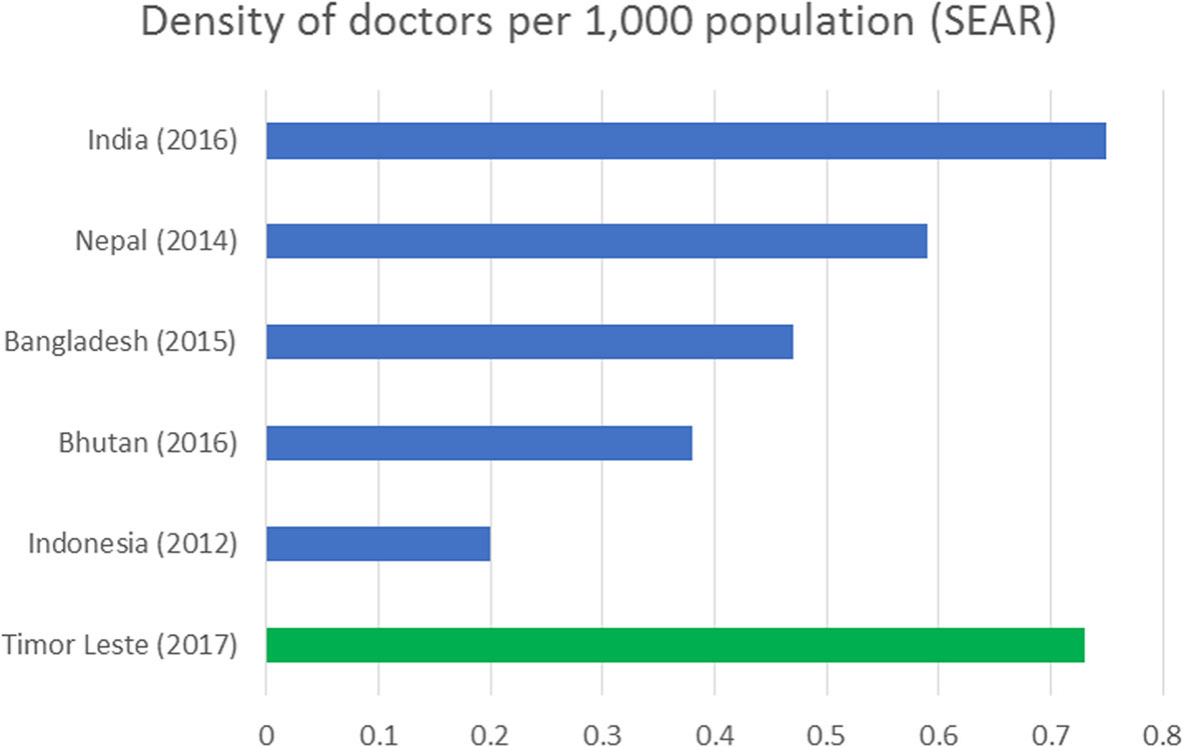
Density of doctors per 1 000 population in the South-East Asia Region. Source: WHO Global Health Observatory data repository (http://apps.who.int/gho/data/view.main.92100)

By mid-2018, the Cuban-supported programme had graduated 934 Timorese medical doctors (KII). Based on the MoH database, this rapid scale-up of the medical workforce has increased the density of doctors per population from 0.03 to 0.71 per 1 000 in 2000 and 2017 respectively, among the highest in South-East Asia (Fig. 2). In 2017, the gender distribution of the medical workforce was almost equal with a male to female (M:F) ratio of 1.01 although the balance was in favour of males at specialist level (M:F ratio of 2.6).

### Relapse into violence, stabilisation and expansion period (2006–2012)

The relapse into violence in 2006 and the tense political environment affected the state’s legitimacy [40] and led to a degradation of accountability and transparency with increased perception of corruption and nepotism [41]. This probably influenced recruitment practices for the health workforce. For example, it is possible that the settlement of the dispute between government and ex-FALANTIL soldiers led to the provision of privileges for the families of veterans of the armed struggle. These privileges included the possibility of obtaining scholarships for higher education bypassing the entry requirements and examinations (Decree Law 8/2009), which opened the system to opportunities for discretion and patronage.

From 2011, the responsibility for selecting students into higher education for all health professions was moved to the Ministry of Education (MoEd) (KII). Key informants highlighted the alarming absence of functioning mechanisms for inter-sectoral coordination, which not only left room for discretion and abuse in decision-making, but also had important consequences for the development of the health workforce. Indeed, the fact that decisions about access to training were moved outside the MoH and some groups were exempted from the standard selection procedures had (and continues to have) critical consequences in terms of quality as well as of numbers and profiles of the future health workforce and may determine a mismatch between intake and needs which the MoH has been left to deal with.

The MoH is not involved in any decisions on the number of students. […] There are no mechanisms for coordination between the MoH and the MoEd. This is a little bit very sad. […] If we recruit too much it will be a disaster for our country. (MoH KI)

The tensions between different national institutions also reveal that, from the aftermath of the independence, the decision-making arena has become more crowded. As a consequence, a number of emerging competing agendas and interests made coordination and alignment more difficult. New actors involved in HRH recruitment and deployment practices included not only the MoH, the MoEd and National University of Timor-Leste (*Universidade Nacional Timor Lorosa’e*—UNTL), but also the Ministry of Finance, the Public Service Commission (PSC), the Human Capital Development Fund (HCDF) and, more recently with the attempt to decentralise the deployment process, the Directors of Municipalities.

As the number of actors involved in HRH decision-making increased, so did the official legislation applicable to the civil service in general and to health workers specifically (Table 5). Importantly, key informants stressed that these regulations were often overlooked or weakly implemented. Indeed, in the increasingly fragmented and instable political environment with constant changes in governments, policies were often blocked at approval or before implementation stage. In other cases, funds are lacking to support implementation.

**Table 5:** Key official laws and regulations on HRH recruitment

Law, Decree law, Directive	Content	Implementation/comments
Directive 3/2000 (UNTAET)	Establishing Civil Service and Public Employment Office (CISPE)	Directive 4/2000 later defining the terms of the Civil Service
East Timor Health Policy Framework (June 2002)	Includes one chapter for Human Resources for Health (first HRH policy)	Still considered the overall policy framework for the sector in 2018
Decree Law 5/2003	Organic Statute of the Ministry of Health	Establishment of Department of Human Resources
Law 8/2004	Statute of the Civil Service	Not fully implemented due to lack of resources (KII)
Decree law 14/2004	Health professional practice including the registration for health professionals	In the absence of health professional councils registration of professionals is done at MoH
National Health Workforce Plan 2005–2015 (draft)	Health workforce situation analysis and strategic plan	Prepared but not approved/implemented
Government Resolution 6/2006	Policy on Decentralisation and Local Government. Established the basis for decentralisation to the Municipal level	Supported by 10/2006 establishing the Secretariat for Decentralisation
Revised National Health Workforce Plan 2007–2015 (draft)	Revision of previous National HRH Plan 2005–2011 including workforce projections	Prepared but not approved/implemented
Decree Law 30/2008	Regulation of scholarships to study abroad	As a key element of the plan for human capital development
Decree Law 34/2008	Regulations for recruitment and selection of civil servants (also promotion)	Further developed with DL 12/2009 (see below)
Decree Law 5/2009	Amending 8/2004 on terms for recruitment	The aim is strengthening accountability in recruitment
Decree Law 7/2009	Establishing Public Service Commission	
Decree Law 8/2009	Award of scholarships to children of veterans	
Decree Law 12/2009	Defines the rule for awarding scholarships for higher education and specialisation (terms of bonding agreements)	Ratified later by Decree 38/2012
Decree Law 36/2009	Legal regime for access to higher education	
Decree Law 16/2010	Statute of the Timor-Leste National University	
Decree Law 22/2011	Amendment to DL 34/2008 re. recruitment, selection and promotion of civil servants	More specific regulations about processes and criteria for recruitment, selection and promotion
Timor-Leste Strategic Development Plan 2011–2030	Including HRH recruitment policy directions and minimum staffing norms	Approved and being implemented
National Health Sector Strategic Plan 2011–2030	Includes projections of HRH needs up to 2030	In the absence of an approved HRH Plan these are the figures used
Human Resources Management Manual (PSC)	Detailed procedures including one chapter for recruitment and selection	Approved and being implemented
Orientation 9/2016	Simplified recruitment of 400 health workers	Executive order waiving 400 newly graduated HWs supported by Government from technical tests
Ministerial Diploma 51/2017 *Saúde na Familia* (Family Health)	Establishing the principles and procedures for the introduction of this family-based PHC model	This programme was a Prime Minister’s initiative
National Health Workforce Plan 2018–2022	Projections updated from NHSSP 2011–2030	Drafted and waiting for government approval
MoH Dispatch 07/2018	Internships Regime (*Internato*) for newly graduated health workers beneficiaries of scholarships.	Terms of the agreement to recruit 320 unemployed health workers as interns
Ministerial Dispatch 05/2018	Establishment of Liaison with Cuban (and Chinese) Medical Brigades	To facilitate communication and coordination MoH/C(and CH)MBs

The implementation of the policies is not consistent. One government comes in with their own policy, then suddenly there’s another government with a different policy and that cause the inconsistencies. (MoH KI)

The increasing number of actors involved in HRH processes, their conflicting agendas, the absence or non-respect of formal regulations and the weak management systems all contribute to the weakening of formal institutions and the emergence of informal practices. Over time, the recruitment process appears to be reverting to the previous practices of patronage and discretion and past hierarchies. This was noted by the key informants.

In relation to recruitment for the MoH, we undergo procedures that are not necessarily based upon the principles of meritocracy. […] We observe the issue of corruption, collusion and nepotism, the family approach. Why this is happening? Because, apart from being a post-conflict country, there are also demands for livelihoods. At the same time, we have significant gaps in regulations and procedures that define the recruitment process and therefore it was still dominated by a situation called ‘calling each other’ (*politika bolu malu*). (national KI)

### HRH reforms under fiscal constraints (2013–2018)

The HRH challenges highlighted above have been made starker by the fiscal constraints, which increased in 2012 when oil revenues dwindled and has worsen since 2015 when the reserves in the Petroleum Fund begun to diminish [42, 43]. Yet a World Bank study [44] reported that the health wage bill increased by 344% between 2008 and 2014, mainly due to the absorption of the newly graduated doctors into the public sector (Table 6), despite the fact that doctors’ salaries are not substantially higher than those of other health professionals [45].

**Table 6:** Number of staff by cadre in the public health workforce

Grades/cadres	2005	2010	2015	2016	2017
General grades	356	615	1 289	1 270	1 225
Special grades					
Medical Specialist	0	9	23	23	29
Medical Doctors	40	75	820	835	891
Midwife	274	388	533	586	619
Nurse	820	883	1 139	1 205	1 272
Assistant Nurse	N/A	N/A	237	234	232
Allied Health Professionals	100	316	440	512	630
Total special grades	1 234	1 671	3 192	3 395	3 673
Total all grades	1 590	2 286	4 481	4 665	4 898

Source: MoH HRH Database 2017

In this context, the absorption into the public sector of the growing number of health workers became a pressing problem. Although estimates vary (from 200 to 600), a high proportion of the pool of qualified doctors and other professionals is currently unemployed, due to lack of funds from the Ministry of Finance to recruit them into the civil service. Worryingly, pressure will be mounting over the next few years as the sustained production of health workers will continue and even increased, based on politically pressured student intake. Indeed, beyond the privileges accorded to veterans’ families described above, the introduction in 2016 of a ‘special regime’ to access higher education, following a simpler selection process without technical tests, for families of military and police officers, diplomats, members of Parliament, journalists, elite athletes and students from international secondary schools [46] contributed to this problem. While in principle, only 10% of the annual intake should be recruited through this modality, key informants noted that the system is abused because of the (politically pressured) acceptance of a high number of students through the special regime. For instance, in 2018 more than 65% of students enrolled in Nursing studies, 60% of those in Midwifery and 35% in Medicine went through the ‘special regime’ (Table 7). While the Cuban Medical Brigade (CMB) deliberately kept out of the politics and simply scaled up their training capacity, the choice, as one respondent explained, has obvious long-term consequences for the quality and distribution of the workforce:

**Table 7:** Student intake in the Faculty of Medicine and Health Sciences of UNTL (2004–2018)

Department	New students														
	2004	2005*	2006	2007	2008	2009	2010**	2011	2012	2013	2014	2015	2016	2017***	2018***
General Medicine	26	510	47	15	20	30	–	42	58	38	36	36	35	52 (12)	107 (40)
Nursing					190	70	–	88	69	83	64	20	73	95 (34)	197 (130)
Midwifery					38	99	–	75	68	84	68	22	76	92 (35)	159 (96)
Pharmacy							–				35	25	70	63	135
Nutrition							–					20	64	59	103
Biomedical/Lab.							–							45	120
Total	26	510	47	15	248	199	–	205	195	205	203	123	318	406	821

Source: UNTL Registry

*2005: first mass enrolment for medical education by CMB

**2010: no enrolment of students due to disputes between MoEd and UNTL about UNTL’s accountability in the selection process

***2017 and 2018: Numbers in parenthesis indicate the intake of new students under the Special Regime, clearly surpassing the statutory 10% of the intake (no data available for 2016 or other cadres)

We need to fix the special regime because the veterans’ children have low marks, but they all choose Medicine. […] If they don’t have a good understanding [of Medicine] then they might give the wrong medication or make mistakes, and this is a problem. (national KI)

As an interim measure to address the absorption issue, a dispatch from the Minister of Health (07/2018/I/MS) was issued in January 2018 to recruit 320 recently graduated health workers as interns (*regimen de internato*) with a standard stipend regardless of professional level of $200 (in rural areas) and $150 (in urban locations) for a period of 6 months to be extended if the need persists and the performance of the intern is satisfactory. However, as KIs said, the success of this programme in reducing the tensions is yet to be seen, and there is a widespread perception that the government has been and continues to waste money on the training of medical doctors rather than using data and evidence on the real needs.

The contrast of the current situation with the immediate post-conflict phase could not be starker. As one respondent put it:

At that time, we didn’t have the people, but the money was there. Now it is probable that we have too many people but not the money. (MoH KI)

## Discussion

Our analysis has some limitations. First, the long period of time covered, spanning over almost 20 years, created challenges during data collection, both in terms of locating and accessing documents that may have been lost, as well as contacting key informants, in particular international actors who have left Timor-Leste. Data were also difficult to retrieve due to the weak information systems. Additionally, many interviewees struggled with recall bias. However, the fact that respondents were open about it and distinguished between clear and only vague memories helped us make sense of their accounts. In a sense, even the mental selection between remembered/forgotten could be interpreted as an indicator of the key, most debated and contested areas of decision-making. In line with this, in order to elicit views on issues clearly remembered and to reduce recall bias, the topic guide we developed focused on the most relevant changes or challenges which respondents were asked to reflect on. We also carefully applied triangulation between different key informants as well as with documents and data to ensure the accuracy of the information and the relevance of the analysis.

Throughout the analysis, we have been explicitly reflective on our positionality with respect to the study subject [47]. Some authors (AAG, JM and SP) are ‘insiders’ to HRH policy-making in Timor-Leste and, although in different roles, have been directly involved in the policy changes described. This has advantages in terms of accessing information and actors as well as bringing participant observations to the analysis, but it may reduce objectivity [48]. The presence of ‘outsiders’ among the authors allowed some distance from the study subject, while at the same time data interpretation could be collectively and iteratively reviewed.

The study findings provide critical insights to enhance our understanding of HRH policy development from a policy and political economy perspective, in a post-conflict setting, but also following the developments over the longer period. The analysis allows the identification of the main phases of the policy trajectory during which different drivers played a significant role in shaping policy outcomes. In terms of actors, there was a shift over time as the relevance of external players faded in favour of that of internal actors and institutions. As in other settings during the immediate post-conflict phase [3, 49–51], Western donors and multilateral organisations were essential and influential in the aftermath of the violence to provide financial and technical support to the nascent Republic, including supporting HRH systems. Later, Cuba became a prominent player, and despite their professed disengagement from ‘politics’, their views and approaches (e.g. gender and geographical balance of students selected) undoubtedly influenced policy-making. More recently, the trajectory in Timor-Leste took a different direction to many post-conflict countries, as national actors have emerged to play a more prominent role and their number and influence have increased in an increasingly fragmented political landscape [52]. It is important to note that the shift in actors’ roles is closely related to and driven by structural changes (specific to Timor-Leste’s economic history) and in particular the move from aid dependency in the first phases of the reconstruction, to financial independence due to oil revenues and, more recently, budget constraints related to the diminishing revenues. Over time, the structural features remained informed by the formal and informal institutions (i.e., the rules and norms) which are prevalent in the country and underlie the overall political and economic context.

Agency and structure elements and their interplay influenced the policy processes and outcomes. Initially, the focus was on health workforce recruitment as is often the case [27], which was seen as fundamental not only for its contribution to the health sector but also in relation to the state-building efforts [6]. As in other settings [53], a key emerging issue was the challenge of achieving a balance between the ‘what’ (technical) with the ‘how’ (political) [52] and reconciliation of a technocratic recruitment process based on rules and merit with political, cultural, relational norms and practices.

It appears that the recruitment system created in the aftermath of independence did consider both technical and political aspects and, perhaps because of this, was generally effective and survived over time. The relative success of this first round of HRH reforms could also be linked to specific individuals, their skills and leadership, both among external and national actors. Local elites were quick to organise themselves with enthusiasm and focus [32], also because of their state-building aspiration through HRH recruitment [6], and there was a substantial alignment in their agendas with that of the technical assistants, as also noted by others [36]. For example, the need felt by national decision-makers to distance themselves from Indonesian rule and ‘KKN’ was in line with the technical and meritocratic approach of external advisers. Additionally, the fact that initially there were few actors in the decision-making arena limited the presence of competing agendas and disagreements. However, while this led to the re-establishment of a HRH recruitment system, the fact that the focus of our analysis rests predominantly on recruitment processes and doctors (rather than on other HR management issues or on other health professionals) highlights how these elements were priorities for policy-makers at central level, while other cadres and issues (such as, deployment) remained under-regulated and dealt with at decentralised level, with the lack of overall strategy and clear official policies to enact, as documented also elsewhere [11, 12, 54]. Additionally, policy-makers paid little or no attention to health workers’ job preferences [45, 55] and decision-making remained a top-down exercise.

Later, while the technical foundations of the recruitment system remained in place and were actually strengthened by institutions such as the PSC, the shift to ‘appointment through training’, due to the Cuban-supported training and to the virtually automatic absorption of newly graduated staff, meant a shift of power from the MoH to new actors (such as, MoEd and UNTL) with diverging interests not related to the long-term needs of the health sector, and increased the possibility of rule-bypassing, discretion and patronage.

The tension between competing agendas may have been initially reduced or resolved thanks to the fiscal expansion and to the presence of the CMB and their ‘substitution’ role [56, 57]. However, with decreasing oil revenues, the CMB potentially phasing out and increased political fragmentation, the competing interests are more difficult to reconcile and the tensions appears starker and riskier in the long term, leading to a low-quality workforce, inappropriate skill-mix, geographic maldistribution and financial unsustainability [58]. In the future, it will be essential that policy processes in Timor-Leste lead to more robust, sustainable and better implemented strategies on HRH training and recruitment.

## Conclusions

Our research aimed to provide a long retrospective account of the development of the systems to recruit health workers in post-conflict Timor-Leste. In this study, we explored the policies in place at central level, the main phases and key drivers of their evolution. Our findings point to patterns and elements both of the agency and structure in shaping the policy-making processes and outcomes.

While the influence of international actors was a prominent feature of the initial phase after the conflict, in Timor-Leste, there was an important change as national actors increased in numbers and relevance later on. This shift led to a fragmented institutional landscape with diverging agendas and lack of inter-sectoral coordination, to the detriment of the long-term strategic development of the health workforce and the health sector. Furthermore, a key issue that cuts across all phases is the difficulty in reconciling the technocratic elements of the reforms with the social, cultural and political aspects. Finally, the research component discussed here looks exclusively at processes and dynamics at central level, and finds that they have focused on regulating HRH recruitment and production, which are the most visible aspects for those operating centrally. Further research on issues such as deployment and transfer, motivation, promotions and career paths (unregulated areas at central level) as well as on how implementation practices differ, modify or bypass central regulations, from the perspective of decentralised actors will be important to shed further light on the topic and complement the central-level perspective.

## Additional files

Additional file 1: Summary of key bibliometric characteristics of documents reviewed (PDF 279 kb)

Additional file 2: Standard topic guide for key informant interviews (PDF 393 kb)

Additional file 3: Data extraction template and coding framework for the documentary analysis (PDF 274 kb)

## Abbreviations

CISPE: Civil Service and Public Employment Office
CMB: Cuban Medical Brigade
DHS: Division of Health Services
HCDF: Human Capital Development Fund
HRH: Human resources for health
HRM: Human Resource Management
KI: Key informant
KII: Key informant interview
KNN: Indonesian acronym for ‘corruption, collusion and nepotism’
LAMS: Latin American Medical School
LMICs: Low and Middle Income Countries
MoEd: Ministry of Education
MoH: Ministry of Health
NCHET: National Centre for Health Education and Training
NGO: Non-governmental rrganisation
PSC: Public Service Commission
SEAR: South East Asia Region
UNTAET: United Nations Transitional Administration in East Timor
UNTL: National University of Timor-Leste (*Universidade Nacional Timor Lorosa’e*)

## Footnotes

1 In 2005, following the hurricane that affected Cuba, some of the medical training was moved to Dili where a Faculty of Medicine was established within the National University of Timor-Leste (UNTL), with Cuban teachers. Currently, students complete their training fully in Timor-Leste and nine Timorese doctors are being trained as faculty for pre-service education (KII).

2 While newly graduated doctors are fast-tracked into the public workforce, nurses and midwifes have to sit an exam for the recruitment into the civil service, under the oversight of the Public Service Commission with a full set of mechanisms to ensure meritocracy and fairness (Human Resources Management Manual, PSC 2014).

3 It is interesting to note that district-based quotas apply to medical students and not to other health sciences careers (e.g., nursing or midwifery).
