# Supplementary material for: Understanding HRH recruitment in post-conflict settings: an analysis of central-level policies and processes in Timor-Leste (1999–2018)
Source: Hum Resour Health. 2018 Nov 29;16:66. doi: 10.1186/s12960-018-0325-5 (PMC6263550; doi:10.1186/s12960-018-0325-5)
Supplement: Supplementary file 1 — Summary of key bibliometric characteristics of documents reviewed (PDF 279 kb) [file 12960_2018_325_MOESM1_ESM.pdf]

**Additional file 1**: Summary of key bibliometric characteristics of documents reviewed

*Documents reviewed by year*

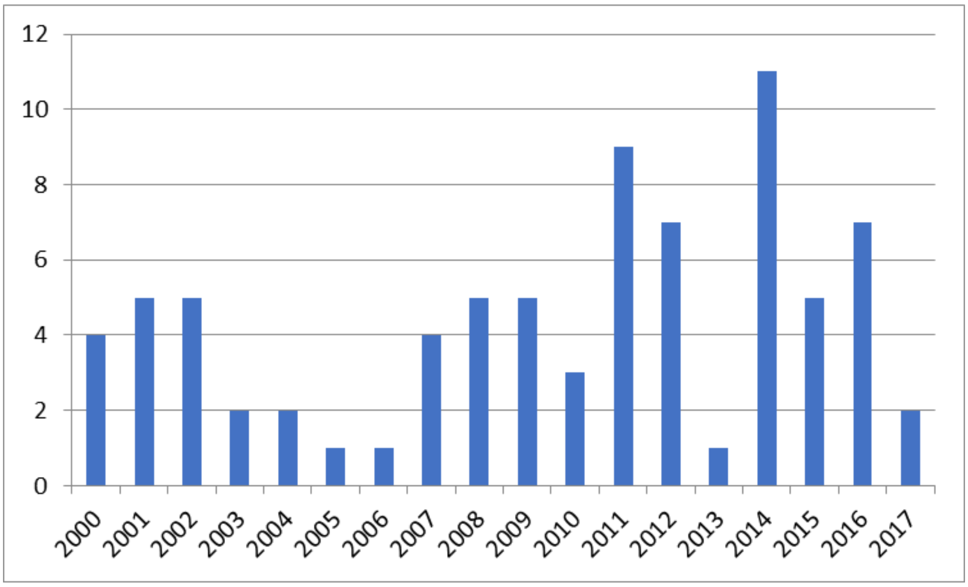


*Documents reviewed by type*

| Type of document | # |
|---|---|
| Decree-Law | 19 |
| Report | 12 |
| Book | 10 |
| Dispatch | 8 |
| Directive | 5 |
| *Others* | 5 |
| Academic paper | 4 |
| Law | 4 |
| Consultancy Report | 3 |
| Policy | 2 |
| Executive Order | 1 |
| Letter | 1 |
| Manual | 1 |
| Presentation | 1 |
