# Supplementary material for: Understanding HRH recruitment in post-conflict settings: an analysis of central-level policies and processes in Timor-Leste (1999–2018)
Source: Hum Resour Health. 2018 Nov 29;16:66. doi: 10.1186/s12960-018-0325-5 (PMC6263550; doi:10.1186/s12960-018-0325-5)
Supplement: Supplementary file 2 — Standard topic guide for key informant interviews (PDF 393 kb) [file 12960_2018_325_MOESM2_ESM.pdf]

Additional File 2: Standard topic guide for key informant interviews

Note details of participants before the interview:

1. Interviewee ID	
2. Date of Interview	
3. Gender	Male ☐ Female ☐
4. Title interviewee	
5. Institution/facility	
6. Region & District (if not national level)	

Introduction

1. Thank participant and briefly re-introduce the study if necessary

[Thank you for agreeing to participate in this study. The purpose of this research is to obtain a better understanding from key informants about the policies relating to health worker recruitment and deployment which were introduced in the post-conflict period, from 1999 until now. We are interested what were the main, most significant changes in policy and practice why they were introduced, how they were implemented, how they are connected and what impact they have had on the health workforce. The experiences you share with us will help us document how recruitment and deployment of health workers have been carried out, what are the challenges and opportunities to propose recommendations to improve these processes in the future.]

2. In order to tailor the interview, if you aren't sure, ask general questions about the role/post of the interviewee, details of organisation s/he works/worked for, how long the person has been in country (as relevant), how long s/he has been in post, and over which period (dates), exact roles and responsibilities in particular with reference to HRH recruitment and deployment, etc., to help with adapting the questions. (this should not take longer than 5 minutes)

1. Context and challenges

Looking back at when you first got involved with the health sector and HRH in Timor-Leste, or at the time of Indonesia's withdrawal (1999) if you've been involved since then, can you describe what was the <u>situation</u> and what were the main challenges concerning the health workforce?

We are particularly interested in the challenges relating to recruitment and deployment of staff, including the forecasting of HRH needs, scholarship assignment, HR selection process, as well as deployment, transfer, and secondment.

Probes:

- Particularly focus on challenges relating to:
    i. Ability to **recruit** enough staff (availability of SOPs/regulations for recruitment?)
        1. Forecast HRH needs
        2. Define appropriate job descriptions
        3. Attract enough students to health disciplines (nursing, midwifery and medicine)
        4. Attract professionals to apply for government positions
        5. Provide scholarships
        6. Ensure incorporation of new graduates to the workforce
        7. Select applicants based on merits
        8. Deal with gender equality during recruitment

    9. Post enough staff (particularly to rural areas)

ii. Ability to **deploy** staff (particularly to rural areas) once they were included in the workforce (availability of SOPs/regulations for deployment?)
    1. Respond to staff requests for transfers
    2. Second staff to respond to service needs (or other needs)
    3. Effectiveness of bonding policies (if these were in place) for successful applicants who were sponsored (keeping them in those positions)

Which cadres were most challenging?

2. Policy responses to challenges

We are now interested in understanding how these challenges were addressed over time in the period between 1999 and 2017. Can you tell us what you think were the main, most significant changes in policy or practice concerning HRH (in particular, need calculation/HRH forecasting, scholarships, recruitment, application, selection processes, deployment and transfer) that were introduced to overcome the challenges?

This includes positive changes but also negative ones, such as 'missed opportunities' when something could have been done, but did not happen.

Note the response of the interviewee:

- Description of main, most significant change/shift in policy or practice: domain/topic of interest, type of change (policy, regulation, informal practice, discussion, etc…), geographical scope (all country or some areas)
- Date/period
- Drivers of the change (or non-change) - see probes below

Probe for changes relating to policy and practice in relation to:

- How HRH needs were calculated and priorities for recruitment identified?
- How was funding for new HRH allocated?
- How decisions were made about the availability of school seats? (this question is for University or MoEd stakeholders); How was that decision coordinated with MoH's identified HRH needs?
- Scholarships to study health disciplines (e.g. criteria for award, criteria for selection of applicants, bonding policies, etc.)
- How did recruitment procedures work and how were they defined (e.g., availability of job descriptions, advertisement of vacancies, application, selection, appointment)? Including how gender equality during recruitment were dealt with.
  - Where these procedures followed in practice (see point 4 below on implementation).
- How did deployment work and how were policies/regulations defined? Think of:
  - Transfer (including exchange)
  - Secondment
  - Bonding (i.e., policies to ensure incorporation of new graduates to the workforce)

  - Where these procedures followed in practice (see point 4 below on implementation).

3. Drivers of changes – for each main policy change/shift

1. What were the main <u>factors</u> which influenced the changes in policy? (Discuss for each of the main, most significant changes described above, in chronological order):
   a. Specific actors/people or organisations? (see below)
   b. Availability of funding?
   c. Political circumstances (elections, newly elected government, ….) or political factors (high-level political support, …)?
   d. Availability of evidence?
   e. Other factors?
2. Who were the main <u>actors</u> (both individuals and organisations) involved in decision-making and implementation around those issues? (ask for each of the main changes):
   a. Who were the main proponents of the change? Were there strong opponents? Why did they support/opposed the change?
   b. What were the mechanisms for coordination between actors and the fora where issues were debated?
   c. Which design features were the most debated?
   d. Who were the 'winners' and 'losers' out of the final decision/change? Why?
   e. How much power and capacity to influence did they have to shape/direct the decisions? What was the power/influence based on (i.e., sources of power and influence, such as (e.g., funding/resources available, influential for historical or political reasons, knowledge holder, politically well linked, etc.), and how was it used?
   f. What was their agenda concerning the issue agendas (i.e. what is their broad aim and ultimate goal, apparent or hidden)?
   g. How did the decision/change impact the rent seeking of key actors?

4. Implementation/operational issues (ask in connection with the previous questions on drivers (e.g., for each policy/change ask about the drivers and then ask about the implementation/operational issues immediately after, before moving to another policy/change)

- How do you think the decision/policy/change was implemented in practice?
  - Would you judge it successful, or not?
  - Would everyone involved agree with you, or not? Why?
- Were HRH practices in line with the (formal) decisions taken and the existing policies? Why, or why not? (give examples)
- What were the most <u>successful aspects</u> of the implementation?
- What were the implementation <u>challenges</u>? Including:

- technical issues (capacity, availability of required inputs/resources, bottlenecks at system level, ...)
- issues with funding and fund flows
- lack of or partial support from key actors
- cultural and contextual features
- economic and political events outside the health sector

- Were they overcome? How? By whom (under whose leadership)?
- What were the main <u>effects/consequences</u> of the change/policy/practice?
  - Did it achieve its objectives? (give specific examples)
  - If so, how?
  - If not, why not?

5. Your recommendations

- What are the lessons which were learned during these experiences?
- Based on these experiences, what do you think should be done to improve the situation regarding recruitment and deployment of health workers?
- Which strategies should be adopted in the future to address the current challenges in these two areas?

Thank you for your time!
(remember to ask for documents related to policies/policy changes named during the interview)
