# Supplementary material for: Understanding HRH recruitment in post-conflict settings: an analysis of central-level policies and processes in Timor-Leste (1999–2018)
Source: Hum Resour Health. 2018 Nov 29;16:66. doi: 10.1186/s12960-018-0325-5 (PMC6263550; doi:10.1186/s12960-018-0325-5)
Supplement: Supplementary file 3 — Data extraction template and coding framework for the documentary analysis (PDF 274 kb) [file 12960_2018_325_MOESM3_ESM.pdf]

Additional File 3: Data extraction template and coding framework for the documentary analysis

Note: Data extraction template: *entire table;* Coding framework: *grey background*

Documents	1	Year
	2	Data extractor
	3	Category/folder
	4	Title of document
	5	Institution/organisation
	6	Type of document (see below)
	7	Source (e.g. personal contact, google search, etc.)
Health workforce issues	8	Situation analysis / challenges for Timor-Leste
	9	Situation analysis / challenges for the health sector
	10	Recruitment (attraction including scholarships, application, selection, appointment, induction)
	11	Deployment (transfer, secondment, bonding, others?)
	12	HRH availability (stock, vacancies, etc.)
	13	HRH accessibility (distribution by district, village, etc.)
	14	HRH acceptability (distribution by gender, age group, ethic group, etc.)
	15	HRH quality (distribution by cadre, academic grade, etc.)
Policy environment	16	Policy objectives and approaches (content of the policy/change)
	17	Actors involved in the policy making and policy implementation processes
	18	Drivers of change (Why did the policy emerge? Who were the main actors involved in decision-making? What are other relevant factors (availability of evidence, funding, …)
	19	Financing of policies (if costs are involved, who is funding the policy?)
	20	Implementation of policies (who was responsible for implementing the policies? What were the main strengths and weaknesses/challenges during implementation?)
	21	Impact of policies (what are the effects of the policy? Does it address the challenges identified? What are the remaining challenges?)
Additional info	22	Other relevant information
	23	Identified gaps (and possible source for the information)
	24	Any other comments
